# YOU’LL SEE: YOUNGER RESEARCHERS INTERVIEWING OLDER PARTICIPANTS

**DOI:** 10.1093/geroni/igz038.2597

**Published:** 2019-11-08

**Authors:** Sarah Jen, Yuanjin Zhou, Mijin Jeong

**Affiliations:** 1 University of Kansas School of Social Welfare, Lawrence, Kansas, United States; 2 University of Washington School of Social Work, Seattle, Washington, United States

## Abstract

In qualitative research, similarities and differences between the participant and researcher influence the research process and dynamics. Specifically, the age difference between older participants and relatively younger qualitative researchers is a common, but under-examined dynamic requiring nuanced, reflexive analysis. Using a life course conceptual framing, this study explored age-related participant-researcher dynamics in interviews from two qualitative studies of older women’s sexual experiences in later life. Participants included 25 women whose ages ranged from 55 to 93 and both studies were completed by the same researcher, a relatively younger woman (age 23 and 28 at times of data collection). A thematic analysis revealed three primary themes: 1) taking care - participants took care of the researcher by offering advice, asking about the researcher’s life, and expressing hopes for a positive future, 2) expertise – varied expertise was demonstrated by the researcher (e.g. substantive and scholarly) and participants (e.g. life experience), and 3) researcher growth - the researcher’s interviewing tactics shifted between the two studies (e.g. use of validation rather than consolation in response to aging-related concerns), indicating a shift in perceptions of aging and later life. Findings indicate that older women participants and younger women researchers are bound together through the life course, by shared gendered experiences, the fact that one will eventually become the other, and the mutual sharing of expertise and caring. Gerontology researchers must actively reflect on the impact of their own identities and aging perceptions on the interviewing process in order to enhance rigor in qualitative research.

